# Effect of doping on the phase stability and photophysical properties of CsPbI_2_Br perovskite thin films†

**DOI:** 10.1039/d0ra08912e

**Published:** 2021-01-05

**Authors:** Lahoucine Atourki, Mari Bernabé, Mohammed Makha, Khalid Bouabid, Mohammed Regragui, Ahmed Ihlal, Mohammed Abd-lefdil, Miguel Mollar

**Affiliations:** MANAPSE Lab, Faculty of Science – Mohammed V University in Rabat 4 Avenue Ibn Batouta BP 1014 RP RABAT Morocco oucine.atourki@um5.ac.ma; Institute of Design & Fabrication (IDF), Polytechnic University of Valencia UPV Spain; Materials Science, Energy and Nano-engineering Department, Mohammed VI Polytechnic University (UM6P) Lot 660, Hay Moulay Rachid 43150 Bengurir Morocco; Materials and Renewable Energies Laboratory (LMER), Ibn Zohr University Agadir Morocco

## Abstract

In this study, we demonstrate that the crystallization process of CsPbI_2_Br films can be modulated when small amounts of additives are added to the precursor solution, leading to the formation of the bright brownish α-phase perovskite films with high orientation along the [100] crystallographic direction. Doped CsPbI_2_Br films exhibit improved crystallinity, with high coverage, large grain size and pinhole-free surface morphology, suitable for making high performance optoelectronic devices. We also explored the role of Cl in the photophysical properties of CsPbI_2_Br perovskite films using the temperature dependent photoluminescence technique. We found that the Cl ions enhance the photoluminescence emission by reducing the density of trap states, and also decrease the exciton binding energy from (22 ± 3) meV to (11 ± 2) meV. We believe this work contributes to understanding the effect of doping on the crystallization process with an in-depth insight into the photophysical properties of the cesium-based perovskite materials.

## Abstract 2

In this study, we demonstrate that in addition to improving the crystallization of CsPbI_2_Br films, the incorporation of Cl^−^ and hydroiodic acid in the precursor solution leads to the formation of films with high coverage and large grains size.

## Graphical abstract

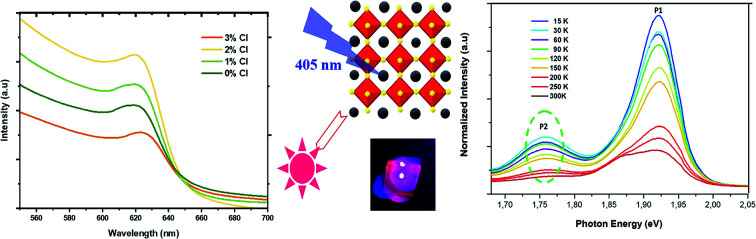

## Introduction

Inorganic cesium-based perovskite materials have gained much research interest for numerous applications due to their excellent thermal stability. The CsPbI_3_ perovskite could be a potential alternative for APbX_3_ (A = MA, FA, GA, X = I, Br),^1^.However, the cubic phase of CsPbI_3_ is only formed at higher temperatures and degrades rapidly in ambient air into a yellow non-perovskite phase.^2,3^ In addition, the low intrinsic thermal stability and the low formation energy of Cs based perovskite materials generate a high density of defects on the surface and grain boundaries of polycrystalline films, limiting their application. The low geometric stability of CsPbI_2_Br is mostly due to the relatively low *t* factor of this compound, compared to that of APbI_3_ (A = MA, FA).^4^ CsPbBr_3_ has been reported to be a very stable perovskite material with high electron mobility.^5,6^ However, its large band gap limits its application as an absorbing layer in perovskite solar cell devices.^7^

One of the promising methods to stabilize the black perovskite CsPbI_2_Br phase at low temperature is the use of chemical additives, such as hydroiodic acid (HI), to improve the solubility of the solute species.^8,9^ Another widely used method is the increase of the surface/volume ratio by synthesizing inorganic perovskite quantum dots^10^ or nanocrystals.^11^ Other studies, dealing with the question of phase transition at low temperature, have used substitution/doping ions to increase the tolerance factor and consequently to increase lattice stability.^12^ Doping has also been demonstrated to be effective in regulating the morphology and passivation of traps^13,14^

Here, we show that the introduction of small amounts of doping elements, provides a new approach to improve the properties and the stability of the functional perovskite phase. In particular, we explore the inclusion of Cl ions into CsPbI_2_Br and examine its effect on the stabilization of the black phase. As found here, the addition of certain amount of CsCl_2_ in a solution of CsPbI_2_Br precursor stabilizes the black phase at room temperature under ambient conditions.

## Results and discussion

**Fig. 1:**
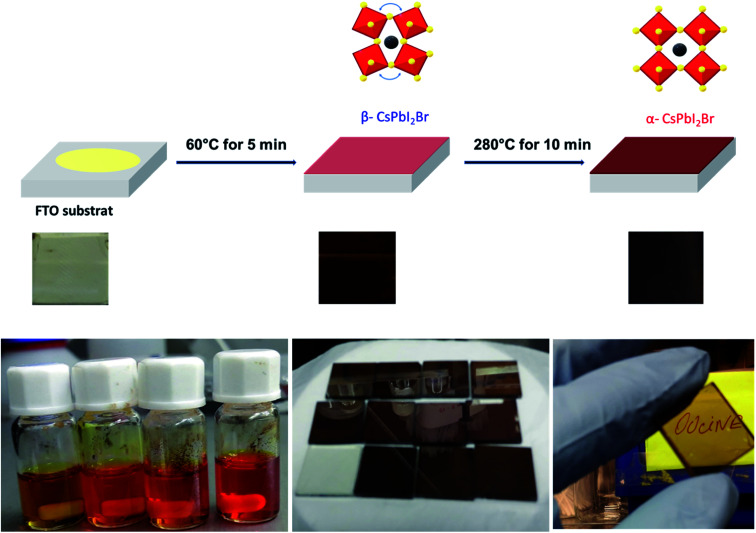
Scheme showing the one spin-coating process of deposition of CsPBI_2_Br thin films and the visual image of the dark brown colored phase of CsPbI_2_Br film synthesized in this work.

**Fig. 2:**
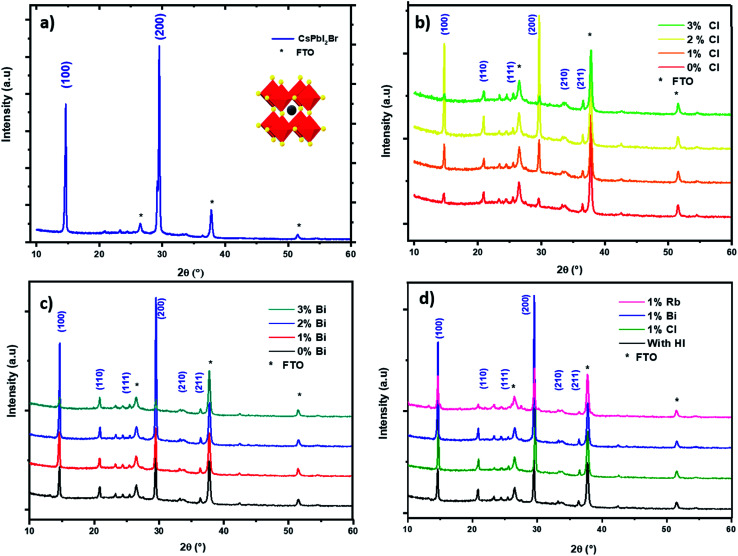
XRD pattern of spin coated CsPbI_2_Br films with different additives: (a) CsPbI_2_Br with HI, (b) with different amounts of chloride, (c) with *x*% of bismuth, (d) XRD pattern of CsPbI_2_Br with different additives.

The pristine CsPbI_2_Br films diffractogram show characteristic perovskite peaks of (100) and (200) located at 14.6° and 29.0°, respectively (Fig. 2a).^15^ The crystal structural details of CsPbI_2_Br are extracted from Rietveld refinement of the XRD pattern. The cubic structure of CsPbI_2_Br is identified as the *pm*3̄*m* space group with a lattice parameter estimated at 6.04744 nm. The XRD pattern of CsPbI_2_Br α-phase corresponds well to the simulated cubic phases of pure iodide (CsPbI_3_) and bromide (CsPbBr_3_), with the corresponding peak positions located between them (see Fig. S1†). Note that the two-steps temperature annealing method used in this work, has a significant impact on the surface coverage of deposited films (Fig. 1).

All films were first annealed at 50–60 °C for 5 min, then the annealing temperature was gradually increased to 290 °C for 10 min. The two-steps annealing method has been optimized to obtain films with high-quality coverage. The freshly spin coated films showed a yellow color indicating the formation of δ-CsPbI_2_Br non-perovskite phase. After gradually increasing the temperature to 60 °C, the films have become brownish-black color. This intermediate phase which forms at low temperature may be the β-phase of CsPbI_2_Br which has a slightly distorted crystal structure compared to the α-CsPbI_2_Br phase (see Fig. S2†). The increase of the annealing temperature to 290 °C for 10 min leads to the formation of a bright brownish α-phase of CsPbI_2_Br with a high orientation along the [100] crystallographic direction (see Fig. S3†).

**Fig. 3:**
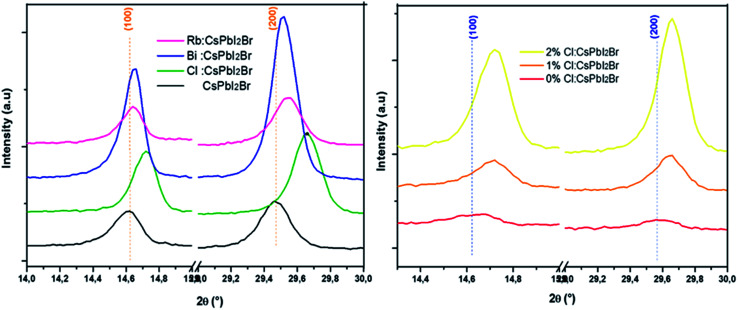
Shift of (100) and (200) XRD peaks of doped CsPbI_2_Br perovskite.

We performed an X-ray diffraction analysis of the CsPbI_2_Br doped films to assess the effect of doping by different elements on the crystal structure (Fig. 2b–d). No binary PbX_2_ (X = I or Br) phase or orthorhombic yellow phase of CsPbI_2_Br could be found. The maximum XRD peak intensities of perovskite films increased with the addition of small amount (2%) of Bi or Cl or HI acid indicating better crystallization. In addition, the XRD peak of doped CsPbI_2_Br (Fig. 3) shows very small shift towards a higher 2*θ* compared to pristine CsPbI_2_Br, indicating a decrease of the crystalline lattice which can be attributed to the smaller ionic radius of Rb^+^ (1.57 Å) and Cl^−^ (1.75 Å) and Bi^3+^ (1.03 Å), compared to Cs^+^ (1.67 Å) and Br^−^ (1.85 Å) and Pb^2+^ (1.19 Å), respectively. Hence, the volumic ratio between the PbX_6_ octahedra and A-site cations decreases when small amounts of Cl^−^ or Bi^3+^ elements are incorporated. This suggests that Cl^−^, Bi^3+^ and Rb^+^ probably replace Br^−^, Pb^2+^ and Cs^+^, respectively, instead of occupying certain interstitial sites. The contraction octahedral volume of PbX_6_ may contribute to stabilizing the black perovskite phase as reported in different studies.^4,16^ In addition, the stronger electronegativity of chlorine (compared to iodine and bromide) in Cl-doped CsPbI_2_Br and the associated more rigid bonds which these form with lead ions lead to the suppression of lattice distortions and therefore a more stable perovskite phase. Furthermore, due to the smaller radius of the dopant elements, the partial substitution leads to an increase in the tolerance factor, which improves the stability. The effective tolerance factor (*t*_eff_) of CsPbI_2_Br (0.855) is slightly larger that of CsPbI_3_ (0.847), but still far away from the values between 0.9 and 1.0 for stable perovskite phase. Hence, the incorporation of doping element may promote the phase stability of CsPbI_2_Br due to the improved *t*_eff_.^20–34^

**Fig. 4:**
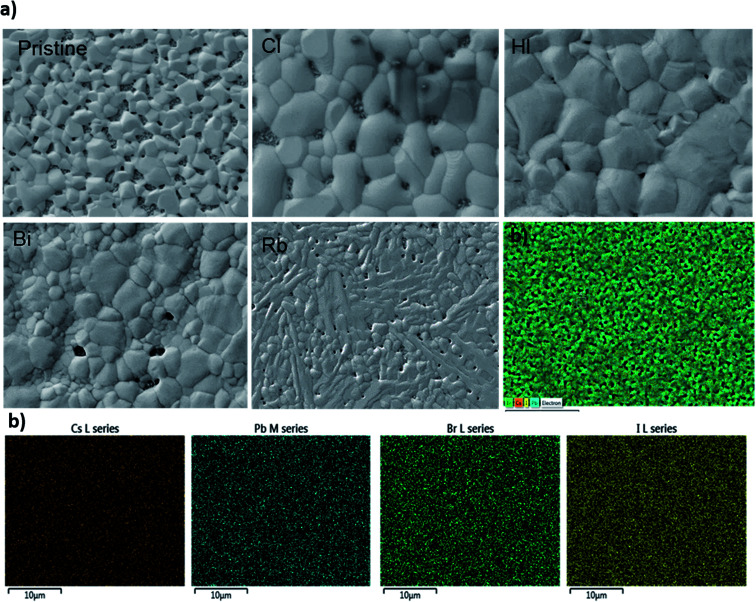
(a) Top-view SEM images of the CsPbI_2_Br film with different additives (b) EdS mapping of CsPbI_2_Br with showing the map distribution of Cs, Pb, I and Br elements. EDS map confirms the uniform distribution of Cs, I and Br elements.

SEM images of the CsPbI_2_Br thin films are shown in Fig. 4 (see also Fig. S4†). The CsPbI_2_Br film exhibits a relatively uniform surface coverage with crystal domains and clear pinholes. Upon incorporation of only 2% of the doping element (Bi, Rb or Cl) or drops of HI, the quality of the film is improved by the increase in the grain size and the disappearance of the pinholes, especially when Cl and HI have been added to the precursor solution. The increase of the grain size with ion doping or HI addition may be related to the modulation of the crystal growth by reducing crystal nucleation and growth rate, leading to a good crystallinity as indicated by the XRD results, and a good surface coverage. The addition of HI, Cl^−^ or Bi^3+^ increases the rate of crystalline grains and suppresses the formation of dense nuclei, hence obtaining larger grains, as shown in Fig. 4.

**Fig. 5:**
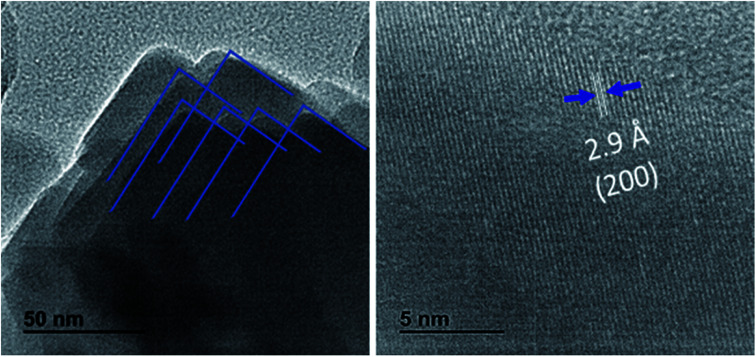
HRTEM Images of CsPbI2Br films. The interplanar spacing of 0.29 nm corresponding to the (200) plane of 3D perovskites was observed.

It has been demonstrated that Cl^−^ ions slow the reaction kinetics of organic perovskites and, therefore, lead to high surface coverage and large grains.^17,18^ On the other hand, herein, HI is reported to first form hydrogen lead iodide (HPbI_3+*x*_) intermediate with PbI_2_, which contributes to the growth of the distorted perovskite phases and therefore leads to high quality films.^19^ A better film quality and large grains means fewer defects and trap states, able decrease the nonradiative recombination at the surface of CsPbI_2_Br. Numerous studies have reported the impact of surface/grain boundary passivation on the reduction of non-radiative loss in perovskites materials (Fig. 5).^20,21^

**Fig. 6:**
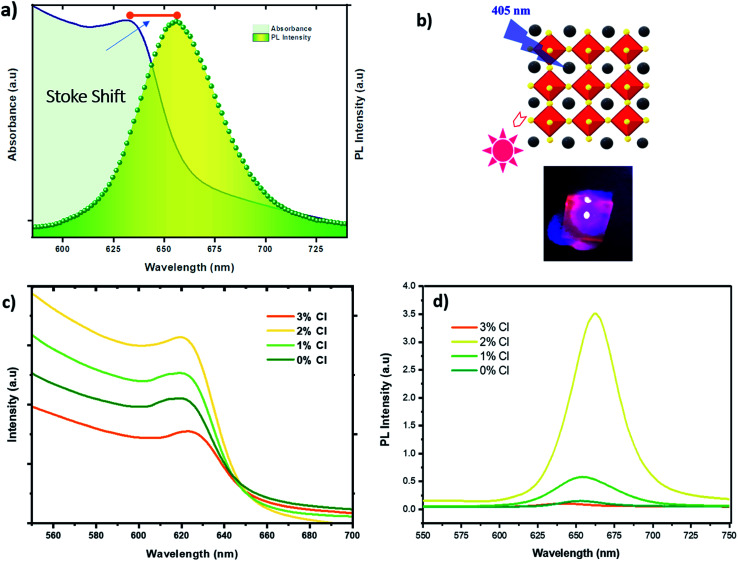
(a) RT absorption and PL spectra of CsPbI_2_Br thin films deposited on FTO substrate (b) scheme of the cubic perovskite crystal structure of CsPbI_2_Br with a photograph of the Red PL emission (c) absorbance spectra of undoped and Cl doped CsPbI_2_Br/FTO samples (d) PL spectra of undoped and Cl doped CsPbI_2_Br/FTO samples.

Trying to elucidate the impact of doping on the photo-physical properties of the CsPbI_2_Br perovskite films, we carried out absorption and photoluminescence measurements at room temperature. The PL spectrum showed in Fig. 6a fits a Gaussian curve well, with a full width at half-maximum (FWHM) is approximately 45 nm, dictated by a homogeneous broadening due to effect of phonon coupling, and corresponding to a highly pure red emission.

The PL emission peak centered at ∼647 nm exhibits a very small Stokes shift between the absorption edge and the PL peak. Different studies have reported the existence of this effect in perovskite materials due to the lattice relaxation (polaronic effects)^23^ and the migration of excitations to low energy sites.^24^ This small observed Stoke shift value indicates an efficient photon recycling, able to significantly improve semiconductor efficiency. Fig. 6c shows that the absorption onset of Cl doped and undoped CsPbI_2_Br of 655–660 nm, indicating an optical bandgap of about 1.87–1.89 eV. The introduction of a small amount of chloride ions does not affect the absorption onset, although, the absorption of the film shows clear improvement when 2% of chloride is incorporated. The 3% Cl:CsPbI_2_Br sample showed a low absorption compared to undoped sample, which might be due to the morphological change induced by the presence of too much chloride and hence the defects introduced by the presence of chloride atoms. Fig. 6d shows an enhancement of the PL intensity with the introduction of 2% of chloride which is in good agreement with UV-vis measurements and XRD results. The enhanced PL emission can be attributed to the surface passivation after chloride incorporation. This result corresponds to the theoretical studies which showed that chloride reduces deep-level defects by substituting Pb–I with Pb–Cl anti-sites, characterized by a higher formation energy with a shallow level.^25^ The incorporation of Cl has also been proven to lead to the passivation of surface defects grain boundaries of hybrid perovskite materials.^26^ It is worth noting that the PL intensity of the CsPbI_2_Br films can be boosted by ∼10 times by changing the environment from vacuum to air (see Fig. S5†). The enhanced PL emission in air can be attributed to the reduced density of trap states, originated from the bromine vacancies in the CsPbI_2_Br lattice. Reducing surface trap states decreases the charge recombination and consequently leads to an increase in the photoelectric efficiency.

**Fig. 7:**
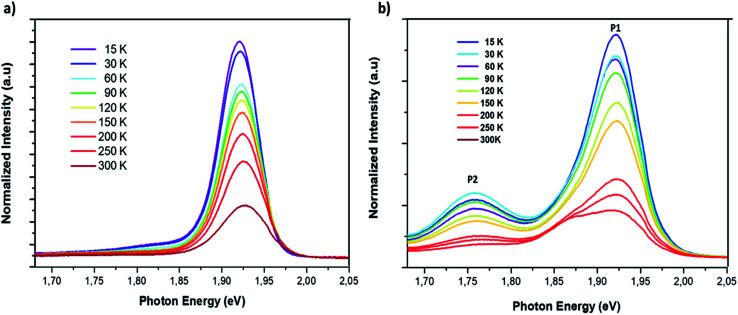
Temperature dependent photoluminescence spectrum of CsPbI_2_Br (a) and 2% Cl doped CsPbI_2_Br (b) with excitation by a 405 nm laser beam.

The study of the photoluminescence properties of perovskite films at low temperatures can provide interesting information about the process of exciton (electron)–phonon interactions and makes it possible to gain depth insights into the crystalline quality, the presence and the nature of defects in the material. Hence, a temperature dependent photoluminescence (TDPL) analysis was carried out for non-doped and Cl-doped CsPbI_2_Br films. Fig. 7 show the evolution of PL intensity of CsPbI_2_Br, with and without chloride, in the temperature range from 12 to 300 K. At room temperature, the PL spectrum display a single dominant peak located at 650 nm. As the temperature decreases to 100 K, a board band located at the low energy side becomes observable and can be attributed to the structural deformation or/and the existence of defects states created by chloride incorporation. The co-existence of multiple PL peaks after chloride incoporation indicates the existence of multiple radiative recombination centers, *e.g.*, intrinsic defect states and extrinsic impurities due to chloride doping.^27,28^ The low energy peak, located at 1.75 eV, may be originated from the emission of trapped excitons. The same behavior has been previously observed in cesium based perovskite.^28^

**Fig. 8:**
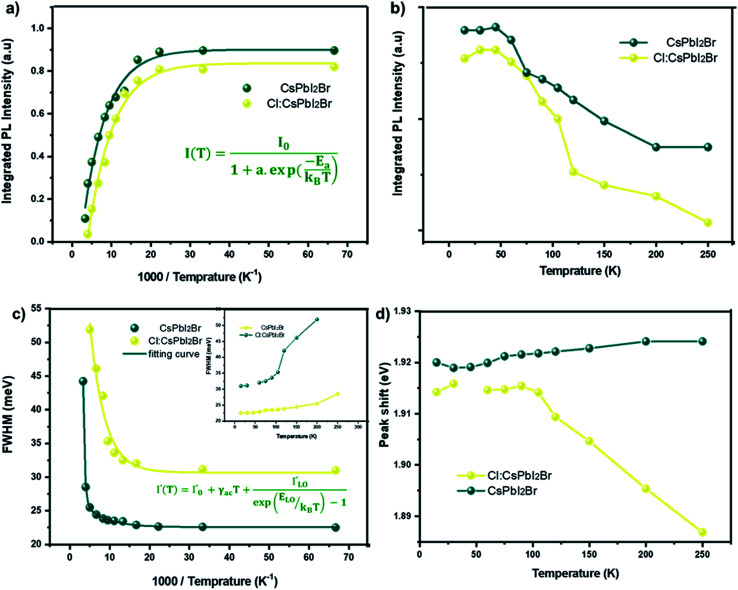
(a and b) Integrated PL intensity as a function of temperature of the CsPbI_2_Br and 2% Cl doped CsPbI_2_Br thin films. The integrated PL intensity was normalized to the initial intensity. (c) Temperature dependent Gauss-fitted full widths at half maximum (FWHMs) and (d) position of the CsPbI_2_Br and 2% Cl doped CsPbI_2_Br thin films.

As the temperature increases from 12 to ∼105 K, the PL spectra of the two samples were slightly blue shifted. In this range of temperatures, the thermal contribution dominates the bandgap variation, while the direct contribution of the electron–phonon interaction is negligible. This trend contrasts with that of typical inorganic semiconductors for which the PL peak shifts with temperature due to the lattice dilatation and the reorientation of the crystallites in the lattice.^29,30^ However, at relatively high temperatures, from 105 to 300 K, the peak position slightly decreases from 1.92 to 1.89 eV for the Cl:CsPbI_2_Br films (Fig. 8d). This phenomenon is largely reported in most semiconductors and is the result of the enhanced electron–phonon interactions caused by the increase in the phonons population.^29^

Fig. 8a and b show the variation as a function of the temperature of the integrated PL intensity of the CsPbI_2_Br and 2% Cl doped CsPbI_2_Br perovskite films. Each PL spectrum has been normalized at its initial PL intensity for easy comparison. The PL intensity as a function of temperature is generally described by the following equation:^31,32^1
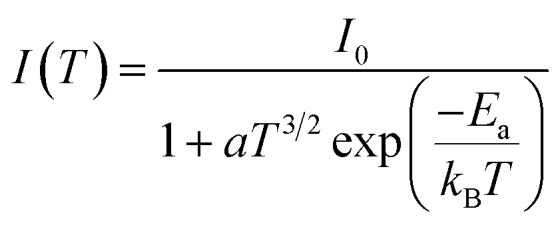
where *I*_0_ is the PL intensity at low temperature limit, *E*_a_ is the activation energy of nonradiative channel, a represents the weight for the probability of nonradiative channel, and *k*_B_ is the Boltzmann constant. The shape of the integrated PL intensity as a function of the temperature is typical of the temperature dependence for semiconductors.^32,33^ At high temperature, the regime of strong thermal quenching, due to thermal exciton dissociation, tends toward a straight-line characteristic of an exponential quenching ∝ exp (*E*_a_/*kT*).^34^ The exciton binding energy extracted was (22 ± 3) meV and (11 ± 2) meV for the undoped and Cl doped CsPbI_2_Br films, respectively. These values are slightly smaller than the value of *E*_a_ reported elsewhere.^35,36^ This difference may be originated from the vibrations of the lattice at relatively high temperature, or from an additional thermal quenching process. It is worth noting that the exciton binding energy values were extracted from the low temperature PL data. The reduced exciton binding energies of the inorganic perovskite compared to other semiconductor,^37,38^ emphasizes their beneficial properties for energy conversion devices due to enhanced rate of exciton separation. It should also be noted that the exciton binding energy of perovskite material is very sensitive to the details of synthesis, composition, and structure.

Additional information on the influence of temperature on the PL emission are provided by the temperature-dependent Gauss-fitted full widths at half maximum (FWHMs), of the undoped and Cl doped CsPbI_2_Br thin films (Fig. 8c). The analysis of broadening of the emission dependent on the temperature was used to investigate the mechanisms of electron–phonon coupling is different materials.^39^ The FWHM as a function of temperature can be generally described by the following equation:

2 *Γ*(*T*) = *Γ*_0_ + *Γ*_ac_ + *Γ*_LO_

3 *Γ*(*T*) = *Γ*_0_ + *γ*_ac_*T* + *γ*_LO_*N*_LO_(*T*)

where *Γ*_0_ represents FWHM at 0 K, *Γ*_ac_ and *Γ*_LO_ are caused by the exciton–phonon coupling with the acoustic and longitudinal optical phonon. *k*_B_ is Boltzmann constant, *γ* represents exciton–phonon coupling strength.^39,40^ The *N*_LO_(*T*) is the Bose–Einstein distribution function, it describes the occupation numbers of the respective LO phonons, given by the following equation: 4
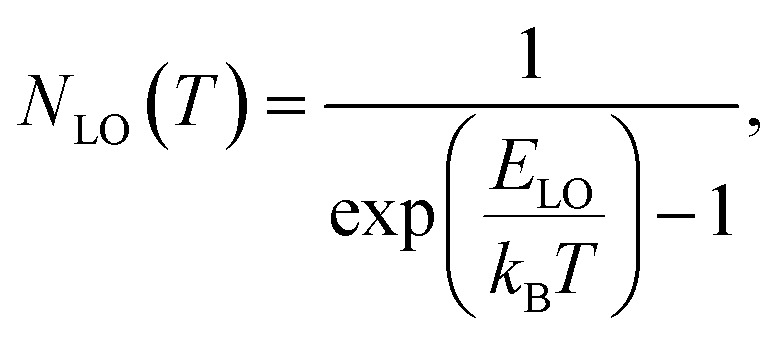
where the *E*_LO_ is the phonon energy.

The values extracted for both films are listed in Table 1. The experimental FWHM values, extracted using the Gaussian best fit of the spectra, and the fitting results clearly show that the FWHM increases with temperature. At low temperatures, the contribution from *Γ*_0_ is larger than that of *Γ*_ac_ and *Γ*_LO_, which is attributed to the increased exciton–phonon interactions.^40–43^ At relatively high temperature, *Γ*_ac_ and *Γ*_LO_ dominates the left side of the formula. The shape of the graph indicates that Frohlich coupling between exciton and LO phonons is stronger, and the peak width increases exponentially with temperature. The extracted parameters listed in Table 1 imply a strong exciton phonon interaction, which agrees with the literature.^44,45^

**Table 1:** Extracted PL linewidth broadening parameters

Sample	*Γ* (meV)	*γ*_ac_ (meV K^−1^)	*E*_LO_ (meV)
CsPbI_2_Br	22.6	14.0	81.0
Cl:CsPbI_2_Br	33.0	25.3	34.4

**Fig. 9:**
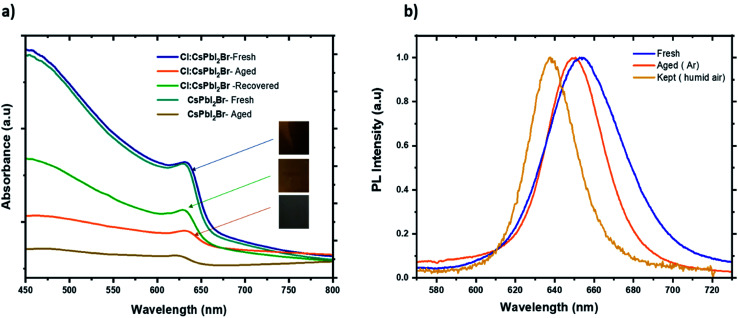
(a) Absorbance spectrum of fresh, aged, and recovered Cl:CsPbI_2_Br thin film. (b) PL spectra of fresh, aged samples of Cl:CsPbI_2_Br films kept in argon and in a humid air.

Finally, the air stability of the prepared films was evaluated without any encapsulation. The films were exposed to ambient air at room temperature and under approx. 55% relative humidity. Fig. 9 shows photographs of the fresh and aged films. The water molecules induced a slow phase transformation from the black perovskite phase to the non-perovskite yellow phase after 7 days in ambient air. The degradation process began at unique sites on the surface of the films before taking up the entire surface. It is well known that at the microscopic level, the water molecules present in the air interacts with the perovskite materials over the grain boundaries, which leads to the dissociation of the cubic perovskite structure to form a yellow non perovskite phase, which is more stable at low temperature. With the introduction of Cl^−^ ions, the perovskite grains become larger, which reduce the grain boundaries and therefore prevents the entry of moisture into the CsPbI_2_Br film. Furthermore, doping has been reported to induce slight distortion of the perovskite crystal structure by creating iodine and bromide vacancies. These additional vacancies could induce lattice strain in the crystal, showing a positive effect in stabilizing the perovskite crystal structure.^46^ It is worth noting that all films kept at room temperature inside the glove box (box with <2 ppm of O_2_ and H_2_O) retain the brownish color, suggesting that moisture is the main catalyst for the perovskite phase transformation.

## Conclusion

In summary, we have shown that small amounts of dopants provide an effective means of improving the morphology and stability of all inorganic CsPbI_2_Br perovskite materials relevant to photovoltaics applications. In particular, we have found that the crystallization process of CsPbI_2_Br films can be modulated when Cl^−^, Bi^3+^ or Rb^+^ ions were added to the precursor solution, leading to the formation of the bright brownish α-phase with high orientation along the [100] crystallographic direction. We also showed that in addition to improving the crystallization of CsPbI_2_Br films, the incorporation of Cl^−^ and hydroiodic acid in the precursor solution leads to the formation of films with high coverage and large grains size. By using the TDPL analysis, we have also found that Cl^−^ ions improve the PL emission by reducing the density of trap states. The exciton binding energies extracted values are (22 ± 3) meV and (11 ± 2) meV for the undoped and Cl doped CsPbI_2_Br films, respectively. We believe this work contributes to understanding the effect of dropping on the crystallization process with an in-deep insights into the photophysical properties of the cesium-based perovskite materials, which offer strategies for designing stable and high performance CsPbI_2_Br-based thin film photovoltaic devices.

## Experimental section

### Preparation of the substrate

The FTO substrates were cleaned with 2% Hellmanex solution, acetone and ethanol under sonication for 15 min, separately.

### Perovskite solution preparation

A 0.6 M CsPbI_2_Br perovskite precursor solution was prepared by stoichiometrically mixing 259.8 mg CsI, 183.5 mg PbBr_2_ (TCI) and 230.5 mg PbI_2_ (TCI) in DMSO : DMF (*v* = 4 : 1) solution under an inert atmosphere in an argon glove box with <2 ppm of O_2_ and H_2_O. The solution was heated overnight at 60 °C to form a clear mixture. The doped CsPbI_2_Br perovskite precursor was prepared by adding small amount of PbCl_2_ or RbI_2_ or BiI_3_. All chemical products were purchased from Sigma Aldrich.

### Perovskite films deposition

The films were deposited, inside the glove box, using spin coating process at 5000 rpm for 30 s. An optimized two steps annealing procedure was used: deposited films inside the glovebox were firstly annealed at 50–60 °C for 5 min. Then, the annealing temperature was increased gradually to 290 °C for 10 min. Finally, the samples were kept in the glove box before analysis.

### Perovskite films characterization

X-ray diffraction patterns of perovskite films coated on ITO substrates were obtained using a RIGAKU Ultima IV in the Bragg–Bentano configuration, using Cu K-alpha radiation (*λ* = 1.54060 Å). The surface morphology of perovskite films was observed by an environmental scanning electron microscope FESEM (Quanta 200 - FEI). The absorption spectra were measured at room temperature using spectrometer Ocean Optics HR4000 equipped with a Si-CCD detector. The integrating sphere was used to collect specular and diffuse transmittance. Room temperature photoluminescence (RPL) measurements were performed using a He–Cd laser source emitting at 325 nm and a back-thinned Si-CCD detector Hamamatsu detected the PL emission.

## CRediT authorship contribution statement

Lahoucine Atourki: conceptualization, methodology, validation, formal analysis, investigation, writing – original draft, project administration. Mohammed Makha: validation, investigation, writing – original draft, writing – review & editing, visualization. Khalid Bouabid: validation, investigation, writing – review & editing, visualization. Bernabe Mari: methodology, validation, formal analysis, investigation, writing – review & editing, visualization. Mohammed Regragui: validation, investigation, writing – review & editing, visualization. Ahmed Ihlal: conceptualization, validation, investigation, writing – review & editing, visualization. Mohammed Abd-lefdil: conceptualization, validation, investigation, writing – original draft, writing – review & editing, visualization, supervision. Miguel Mollar: conceptualization, methodology, validation, formal analysis, investigation, writing – review & editing, visualization, supervision.

## Conflicts of interest

The authors declare that they have no known competing financial interests or personal relationships that could have appeared to influence the work reported in this paper.

## Supplementary Material

RA-011-D0RA08912E-s001
