# Supplementary material for: Effect of doping on the phase stability and photophysical properties of CsPbI2Br perovskite thin films
Source: RSC Adv. 2021 Jan 5;11(3):1440–9. doi: 10.1039/d0ra08912e (PMC8693609; doi:10.1039/d0ra08912e)
Supplement: RA-011-D0RA08912E-s001 [file RA-011-D0RA08912E-s001.pdf]

Electronic Supplementary Material (ESI) for RSC Advances.
This journal is © The Royal Society of Chemistry 2020

Effect of doping on the Phase Stability and Photophysical Properties of $CsPbI_2Br$ Perovskite Thin Films

L. Atourki[1], M. Bernabé[2], M. Makha[3], K. Bouabid[4], M. Regragui[1], A. Ihlal[4], M. Abd-lefdil[1], M. Mollar[2].

[1] MANAPSE Lab, Faculty of Science – Mohammed V University in Rabat, 4 Avenue Ibn Batouta BP 1014 RP, RABAT Morocco
[2] Institute of Design & Fabrication (IDF) – Polytechnic University of Valencia UPV, Spain
[3] Materials Science, Energy and Nano-engineering Department, Mohammed VI Polytechnic University (UM6P), Lot 660, Hay Moulay Rachid, 43150 Bengurir Morocco.
[4] Materials and Renewable Energies Laboratory (LMER), Ibn Zohr University, Agadir, Morocco

Corresponding author: Oucine.atourki@um5.ac.ma

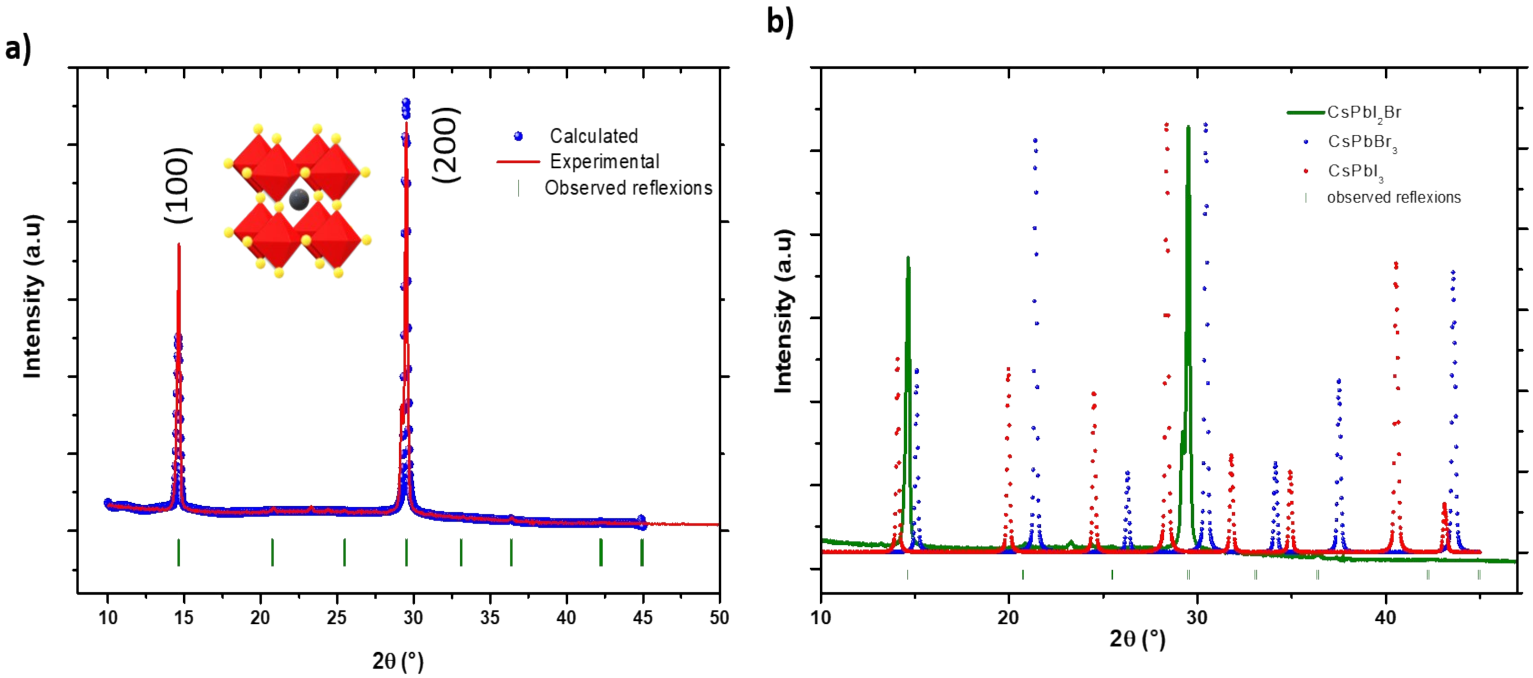

S.1 a) Experimental and calculated XRD pattern of the spin coated $CsPbI_2Br$ films **b)** Experimental XRD pattern of $CsPbI_2Br$, $CsPbI_3$ and $CsPbBr_3$.

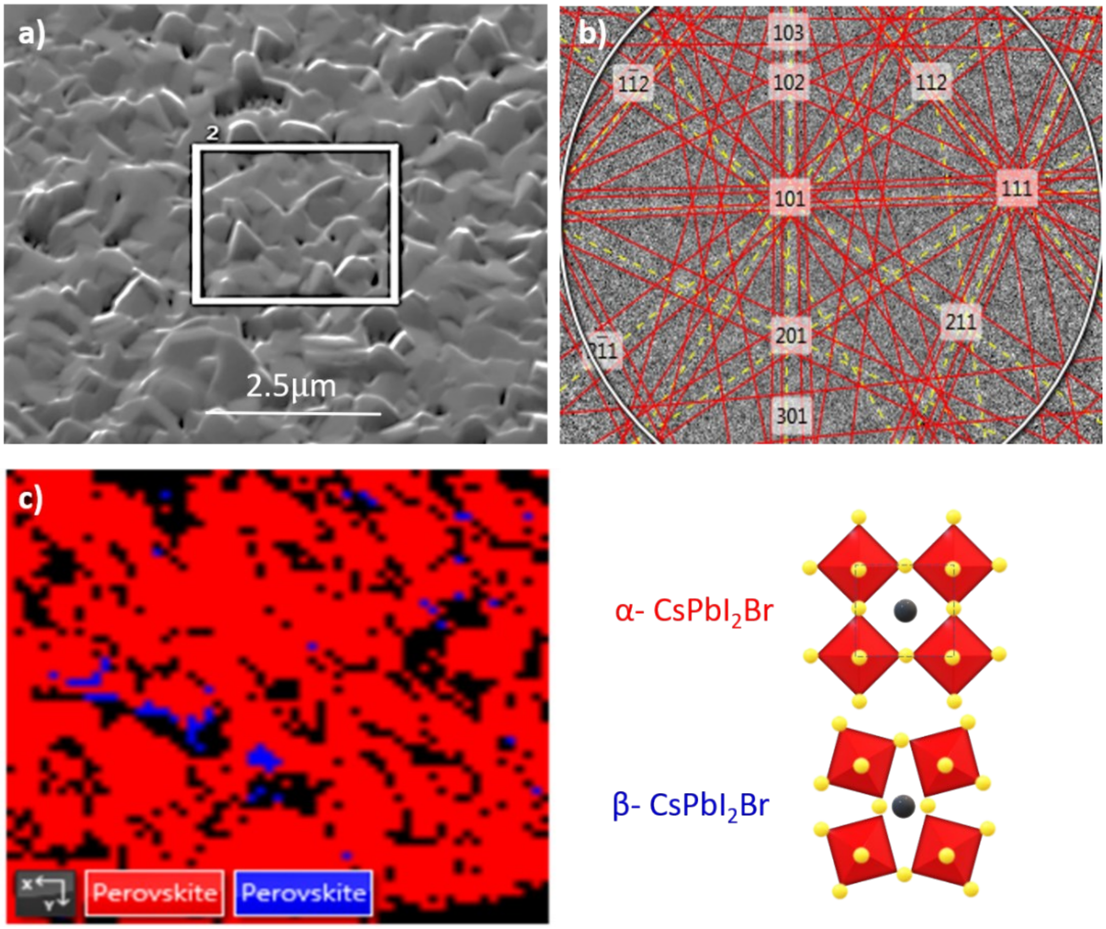

S.2 a) Scanning electron microscope (SEM) image of $CsPbI_2Br$ thin film tilted at 70 **b)** representative sharp Kikuchi diffraction lines of $CsPbI_2Br$ thin film collected in traditional electron backscatter diffraction (EBSD) geometry at 20 kV accelerating voltage **c)** Inverse pole figure (IPF) map generated from EBSD of CsPbI2Br thin film with IPF color key of the two perovskite phases α-$CsPbI_2Br$ and β-$CsPbI_2Br$

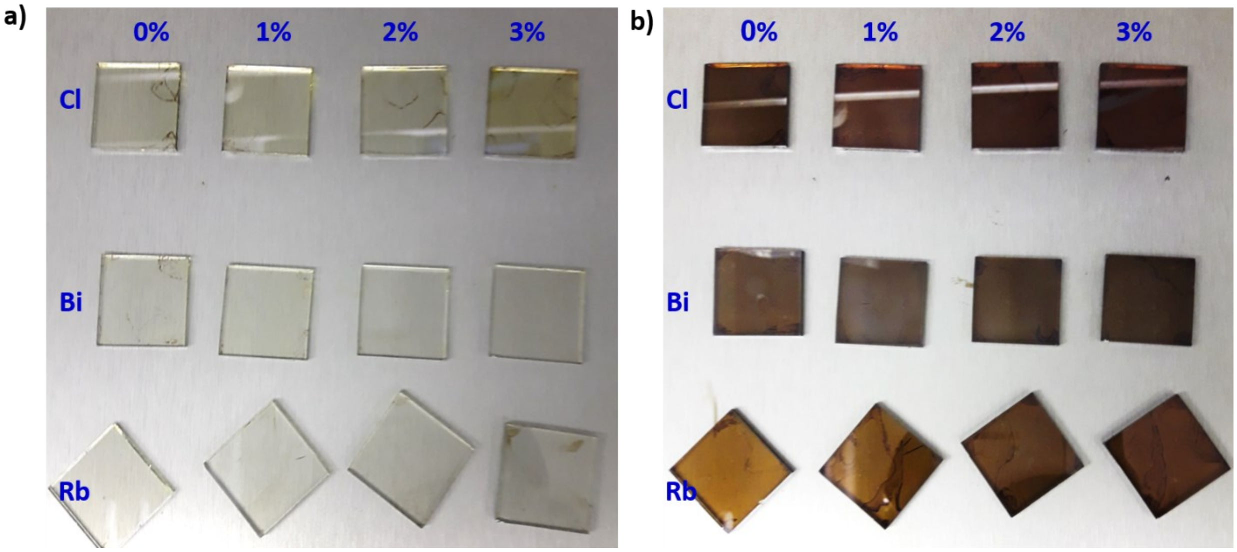

S.3 The visually image of dark brown colored phase of $CsPbI_2Br$ films synthesized in this work during different phases of thermal annealing: **a)** After 1 min at 60 ºC, **b)** after 10 min at 180 ºC.

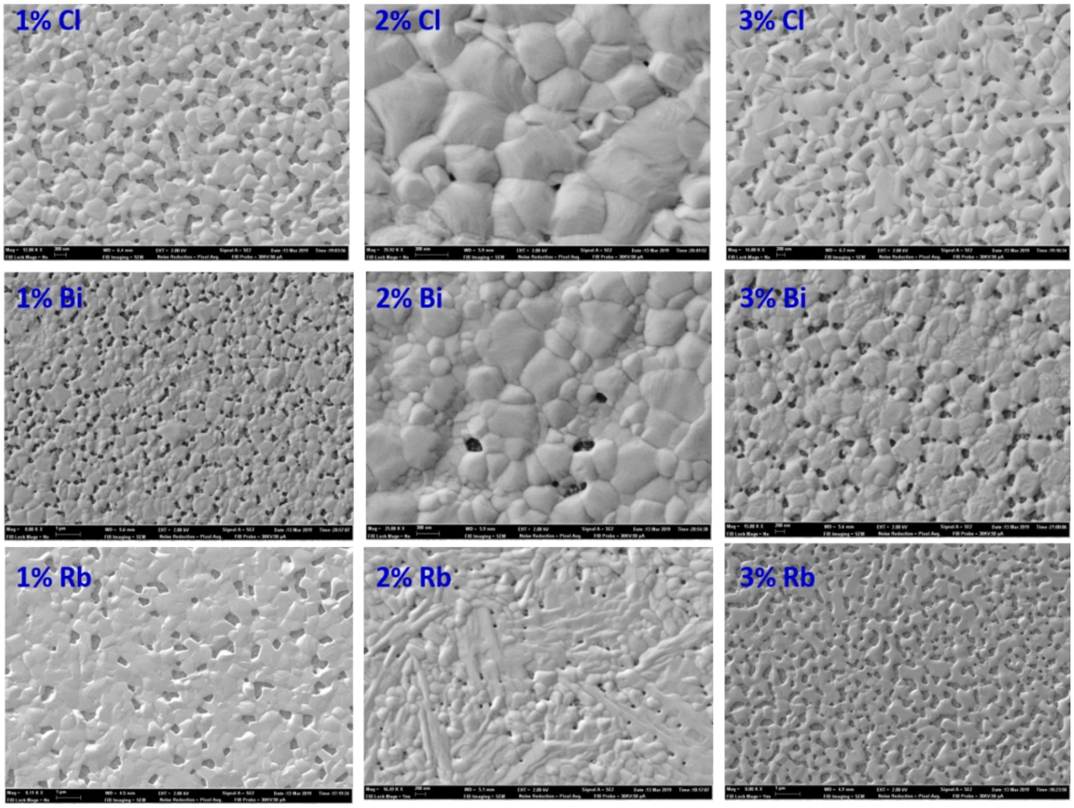

S.4 SEM images of different $CsPbBr_2I$ doped with Cl, Bi and Rb with different concentration

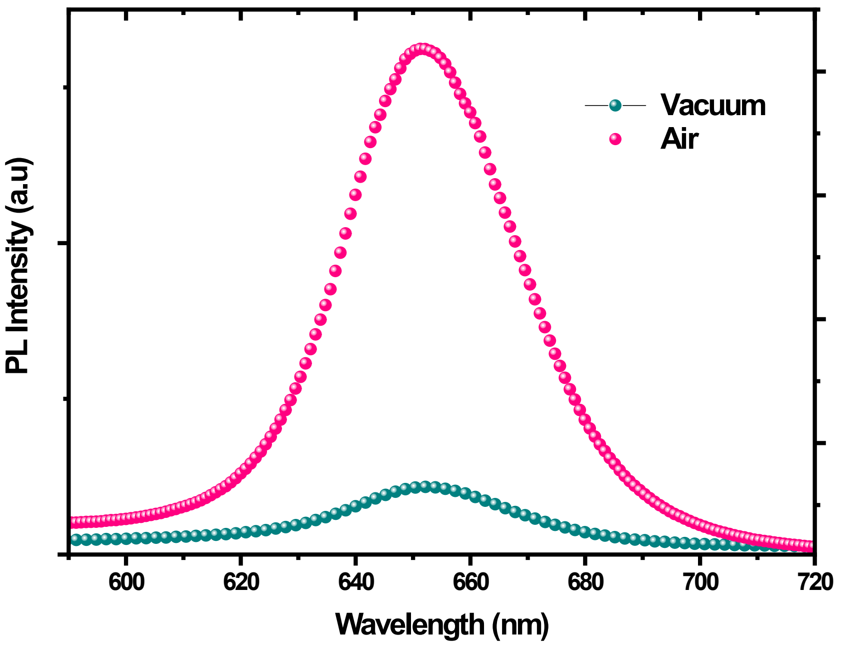

S.5 PL spectra of Rubidium doped $CsPbI_2Br$ films with different Rb percentage.

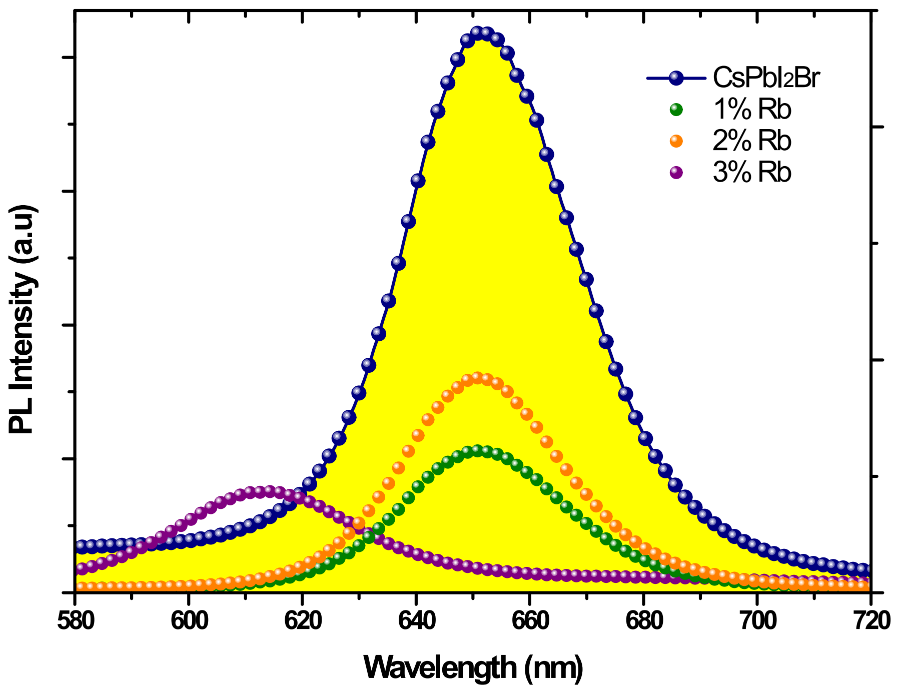

S.6 PL spectra of $CsPbI_2Br$ films with different in vacuum and in ambient air.

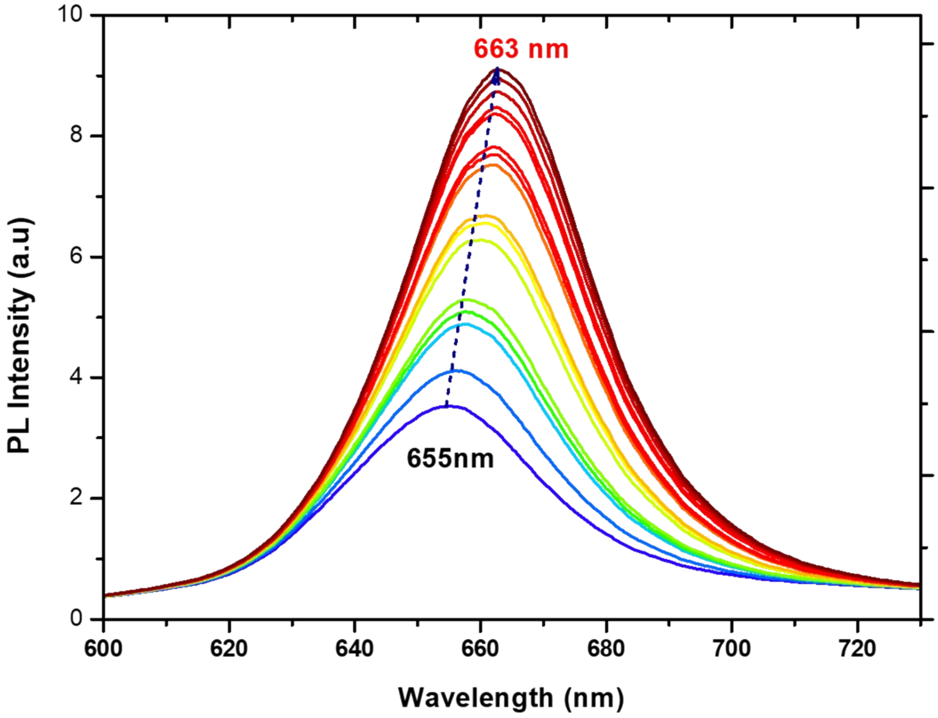

S.7 PL spectrum of $CsPbI_2Br$ film under continuous laser illumination
